# Supplementary figures and images for: A Diffusive Homeostatic Signal Maintains Neural Heterogeneity and Responsiveness in Cortical Networks
Source: PLoS Comput Biol. 2015 Jul 9;11(7):e1004389. doi: 10.1371/journal.pcbi.1004389 (PMC4497656; doi:10.1371/journal.pcbi.1004389)

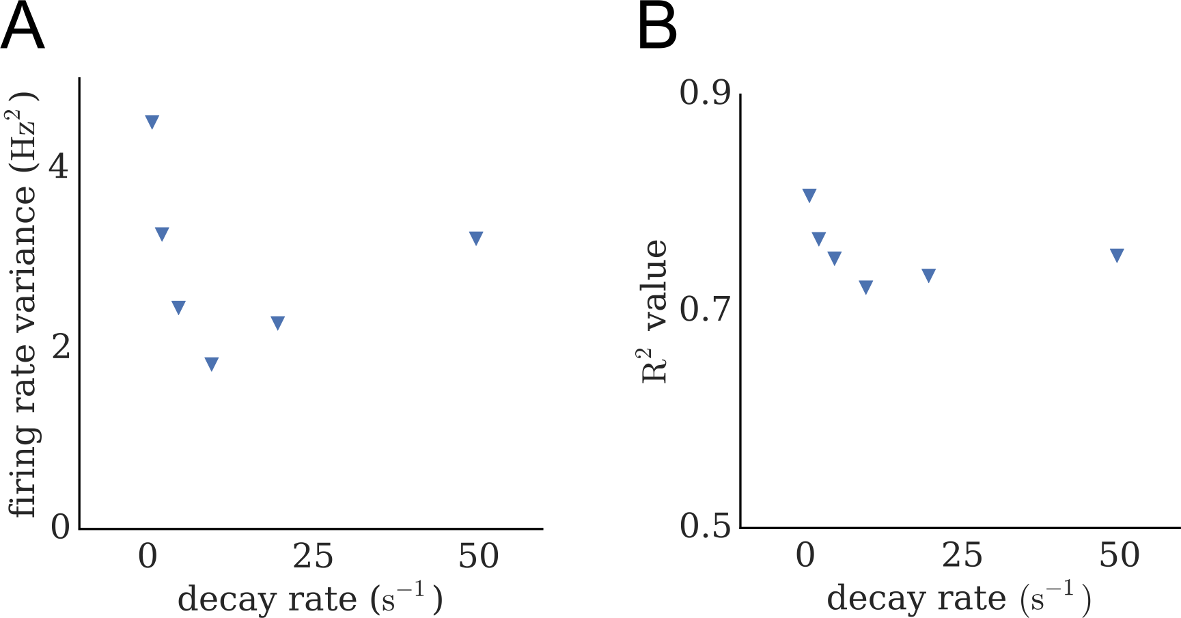

Supplement: S1 Fig — (A) Variance of the steady-state firing rate distribution as the rate of NO decay, λ, is varied. (B) Linearity of the population response to a change of inputs as the rate of NO decay is varied. (TIF) [file pcbi.1004389.s001.tif]

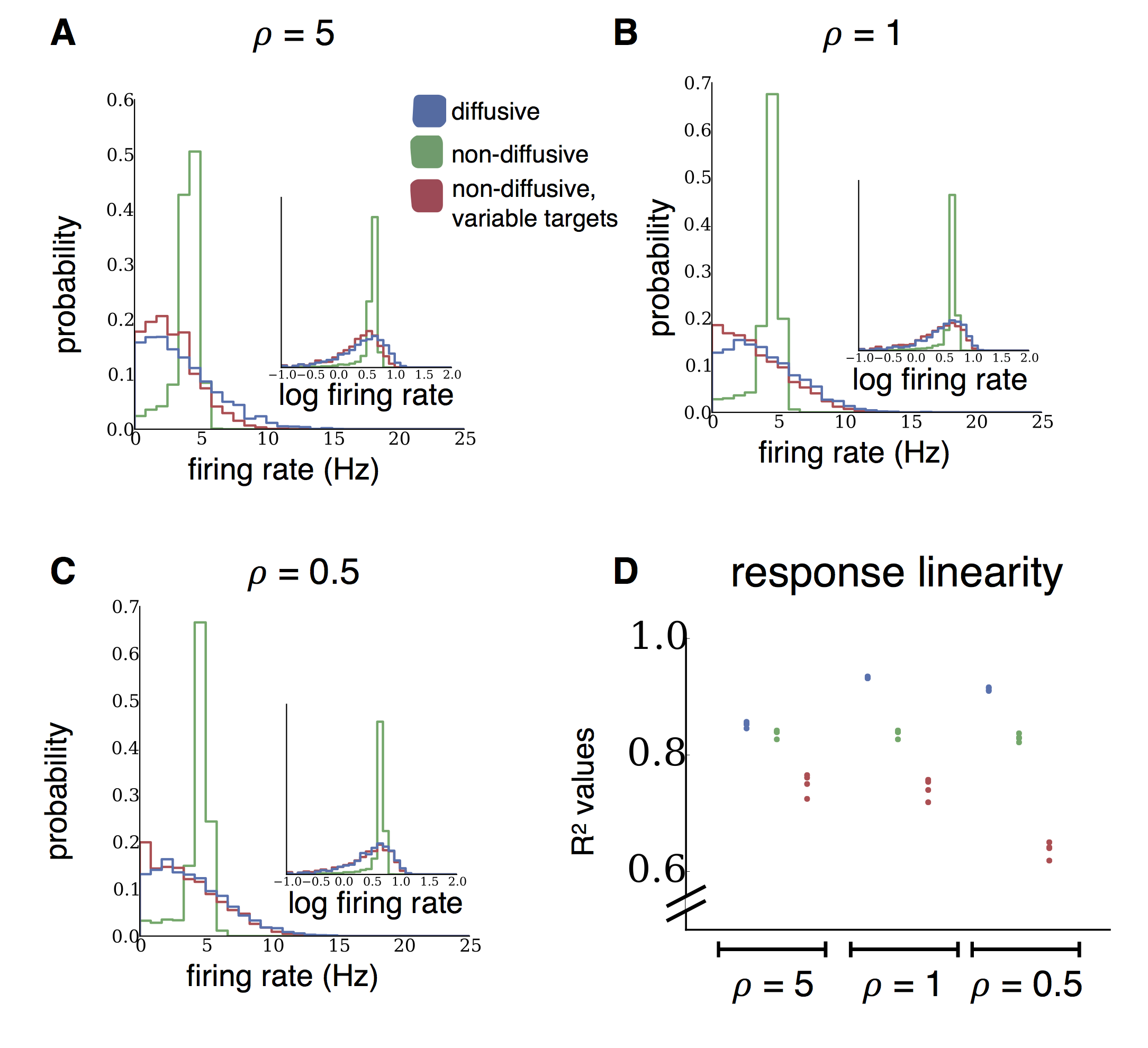

Supplement: S2 Fig — These results qualitatively agree with those for the random networks used throughout the study. The simulations shown here were identical to those in Figs 1D and 5 of the main text, but the connection probability between neurons had a Gaussian shape. (A-C) ρ, the ratio of the connectivity range and the diffusive range, is varied across a wide range of values (ρ = 5.0, 1.0, 0.5). (D) Each point represents the R 2 value of a linear fit as in Fig 5A–5C, for one network. Spatial dependence in the connection probability between two neurons was introduced as follows: Pc(d)=ϵe-d22s2,(14) where d is the Euclidean distance between the neurons and s is a constant defining the connectivity range of the network. 2D positions on the torus are bounded such that x, y ∈ (0, 1). Given a diffusive range of 0.1mm, values for s were therefore set as 0.05, 0.1, and 0.5. The ratio of s and the diffusive range was defined as ρ, which had values of 0.5, 1.0 and 5.0. (TIF) [file pcbi.1004389.s002.tif]
